# Carboranyl-Chlorin e_6_ as a Potent Antimicrobial Photosensitizer

**DOI:** 10.1371/journal.pone.0141990

**Published:** 2015-11-04

**Authors:** Elena O. Omarova, Pavel A. Nazarov, Alexander M. Firsov, Marina G. Strakhovskaya, Anastasia Yu. Arkhipova, Mikhail M. Moisenovich, Igor I. Agapov, Valentina A. Ol’shevskaya, Andrey V. Zaitsev, Valery N. Kalinin, Elena A. Kotova, Yuri N. Antonenko

**Affiliations:** 1 Belozersky Institute of Physico-Chemical Biology, Lomonosov Moscow State University, Moscow, Russia; 2 Biological Department, Lomonosov Moscow State University, Moscow, Russia; 3 Federal Scientific and Clinical Center for Specialized Medical Service and Medical Technologies, FMBA, Moscow, Russia; 4 Shumakov Research Center of Transplantology and Artificial Organs, Moscow, Russia; 5 Nesmeyanov Institute of Organoelement Compounds, Russian Academy of Sciences, Moscow, Russia; Massachusetts General Hospital, UNITED STATES

## Abstract

Antimicrobial photodynamic inactivation is currently being widely considered as alternative to antibiotic chemotherapy of infective diseases, attracting much attention to design of novel effective photosensitizers. Carboranyl-chlorin-e_6_ (the conjugate of chlorin e_6_ with carborane), applied here for the first time for antimicrobial photodynamic inactivation, appeared to be much stronger than chlorin e_6_ against Gram-positive bacteria, such as *Bacillus subtilis*, *Staphyllococcus aureus* and *Mycobacterium sp*. Confocal fluorescence spectroscopy and membrane leakage experiments indicated that bacteria cell death upon photodynamic treatment with carboranyl-chlorin-e_6_ is caused by loss of cell membrane integrity. The enhanced photobactericidal activity was attributed to the increased accumulation of the conjugate by bacterial cells, as evaluated both by centrifugation and fluorescence correlation spectroscopy. Gram-negative bacteria were rather resistant to antimicrobial photodynamic inactivation mediated by carboranyl-chlorin-e_6_. Unlike chlorin e_6_, the conjugate showed higher (compared to the wild-type strain) dark toxicity with *Escherichia coli* Δ*tolC* mutant, deficient in TolC-requiring multidrug efflux transporters.

## Introduction

The medicinal chemistry of carba-*closo*-dodecaboranes (carboranes [1]) has been traditionally centered on their use in boron neutron capture therapy (BNCT) of tumors [2,3]. Based on the known ability of various porphyrin-related photosensitizers to accumulate in tumors and generate cytotoxic reactive oxygen species killing cancer cells, conjugation of carboranes with these compounds was considered as a way to improve both their delivery to tumors and therapeutic efficacy. Actually, such studies resulted in design and synthesis of promising agents for BNCT, photodynamic therapy and fluorescence imaging of tumors [4–12]. On the other hand, in a series of studies carboranes were used as drug pharmacophores [13–15]. So far, no research has to our knowledge concerned antimicrobial photodynamic effect of boronated carboranes, although photodynamic inactivation (PDI) of bacteria has long been studied with different photosensitizers [16–32] resulting in a great variety of medicinal applications. Of note, some data on dark bactericidal and fungicidal activity of 1-(aminoalky)-1,2-dicarba-*closo*-dodecaborane [33] and *o*-carboranylalanine [34] were earlier reported. The cytotoxic efficacy of derivatives of polyhedric boron complexes could be related to their unique binding [35] and membrane-penetrating [36,37] properties, the latter being associated with delocalization of their charge.

Much attention is attracted now to studying antimicrobial PDI (aPDI), because this therapeutic modality, being effective against drug-resistant infections, might allow for the development of a valuable alternative or supplemental option to the current antibiotic-based treatments [26,38]. The major targets of PDI in bacterial cells are still debated, albeit strong evidence has been obtained in favor of damage to outer cell structures being critical [19,26,30,31,39–46]. In fact, various authors have concluded that although DNA damage occurs, it may not be the primary cause of bacterial cell death [47,48].

Among a great variety of photosensitizers studied as agents in aPDT, derivatives of chlorin [20,23,24,49–53] and bacteriochlorin [54] attracted special attention. In particular, Hamblin and colleagues showed that covalent conjugates of chlorin e_6_ with poly-L-lysine [23,24] and polyethyleneimine [51] were efficient photosensitizers (PS) of both gram-positive and gram-negative bacteria, because the polycationic molecular constructs increased binding and penetration of the PS into impermeable gram-negative cells. Chlorin-polyethyleneimine conjugates were also effective in PDI of fungi [54].

Here, we for the first time applied boronated chlorin e_6_ amide (BACE, chlorin e_6_ 13(1)-N-{2-[N-(1-carba-closo-dodecaboran-1-yl)methyl]aminoethyl}amide-15(2), 17(3)-dimethyl ester) [8,9] for aPDI and found this photosensitizer to show much higher efficacy against Gram-positive than against Gram-negative bacteria.

## Materials and Methods

### Chemicals

The sodium salt of 13(1)-N-{2-[N-(1-carba-*closo*-dodecaborane-1-yl)methyl]aminoethyl}amide-15(2),17(3)-dimethyl ester of chlorin *e*_6_ (BACE) was synthesized and described earlier [8,9]. Chlorin e_6_ was obtained from Porphyrin Products (Logan, UT). *E*. *coli* total lipid extract was from Avanti polar lipids (Alabaster, AL). 5(6)-carboxyfluorescein (CF) was from Sigma-Aldrich (St. Louis, MO).

### Carboxyfluorescein leakage from liposomes

Dye-loaded liposomes were prepared by evaporation under a stream of nitrogen of a 2% solution of *E*. *coli* total lipid extract in chloroform followed by hydration with a buffer solution containing 230 mM Tris and 100 mM CF. The mixture was vortexed, passed through a cycle of freezing and thawing, and extruded through 0.1-μm pore size Nucleopore polycarbonate membranes using an Avanti Mini-Extruder. The unbound CF was then removed by passage through a Sephadex G-50 coarse column with a buffer solution containing 10 mM Tris and 100 mM KCl, pH 7.4. To initiate the release of liposome-entrapped CF, the liposomes were incubated in the dark at room temperature with photosensitizers for 5 min and then illuminated with a halogen light source (“NovaFlex”, World Precision Instruments, USA) for 1 min. CF release from liposomes into the bulk solution was monitored by an increase in CF fluorescence resulting from its dequenching upon dilution. Fluorescence of liposomes loaded with 100 mM CF was monitored at 520 nm (excitation at 490 nm) with a Panorama Fluorat 02 spectrofluorimeter (Lumex, Russia). The extent of CF efflux was calculated as (F_t_−F_0_)/(F_100_-F_0_), where F_0_ and F_t_ represent the initial fluorescence intensity and the fluorescence intensity at the time *t*, and F_100_ is the fluorescence intensity after complete disruption of liposomes by addition of the detergent Triton-X100 (final concentration, 0.1% w/w).

### Bacterial strains

Standard laboratory strains *Bacillus subtilis subs*. *subtilis* Cohn 1872, strain BR151 (*trpC2 lys-3 metB10)*, and *E*. *coli* Castellani and Chalmers 1919, strain W3110 (*F* ^-^ *lambda-IN(rrnD-rrE)1 rph-1*) were used in this study. *Staphyllococcus aureus* Rosenbach 1884 (entry #144) and *Mycobacterium sp*. (entry #377) were obtained from the Microorganisms Collection of the Moscow State University. The deletion strain JW5503 (ECK3026 in the Keio collection [55], the *E*. *coli* Δ*tolC* mutant), devoid of the *tolC* gene, was kindly provided by Hironori Niki, National Institute of Genetics, Japan [55].

### Bacteria growth

Bacterial cells were grown at 37°С in LB medium at 140 rpm shaking frequency. Overnight culture was diluted in fresh growth medium and grown to mid-log phase, then washed twice in sodium phosphate buffer (pH 7.4) at 7,000 rpm for 5 min and resuspended to an optical density of about 0.8 at 600 nm, corresponding to approx. 10^8^ cells/ml. The resulting bacterial suspension was used for further experiments.

### aPDI measured by plating

To measure aPDI, we used the method of serial dilutions. Suspensions of bacteria were incubated in the dark at room temperature for 10 min with 1 nM—10 μM chlorin e_6_ or BACE in sterile PBS and illuminated with red light (λ > 630 nm) obtained with KS-15 filter from a halogen light source (“NovaFlex”, World Precision Instruments, USA). The survival of bacteria was assessed through colony forming unit (CFU) counts. CFU were determined by bacterial plating on Petri dishes of serial dilutions. The fraction of survived cells was calculated as the ratio of CFU of bacteria illuminated in the presence of a certain concentration of a photosensitizer to CFU of the control bacteria (grown in the absence of photosensitizers in the dark).

Alternatively, to avoid serial dilutions, suspensions of bacteria (2–5*10^4^ cell/mL) were incubated in the dark at room temperature for 10 min with 1 nM—10 μM chlorin e_6_ or BACE in nutrition broth. 0.2 mL of the bacterial suspensions was placed in a 96-well plate and illuminated with red light, as described above. The viable bacteria were assessed through CFU counts. The CFU for bacteria grown in the dark in the absence of photosensitizers was taken as 100% (control).

### Dark toxicity of BACE and chlorin e_6_ measured by optical density

Overnight *E*. *coli* bacterial cells cultures were diluted in fresh LB media. Chlorin e_6_ or BACE (100 nM—50 μM) were added to bacterial cultures (1–5*10^6^ cells/ml), placed in 96-well plates, Cell density was determined by absorbance at 600 nm using an Multiscan FC multimode reader (Thermo Scientific, USA) after bacteria were allowed to grow in the dark within 21 hours.

### Accumulation of photosensitizers by bacterial cells

Accumulation of photosensitizers by bacterial cells was evaluated from the fluorescence of the pellet obtained after centrifugation at 12000g for 2 min. Cells were incubated for 10 min in the dark with the indicated amount of a photosensitizer. The pellet obtained after centrifugation was treated with 0.1 M NaOH / 1% SDS. The photosensitizer concentration was determined from its fluorescence by using a calibration curve for the solutions of different concentrations in 0.1 M NaOH / 1% SDS.

### Fluorescence correlation spectroscopy

Fluorescence correlation spectroscopy (FCS) measurements were carried out with a home-made FCS setup [56,57] including an Olympus IMT-2 inverted microscope with a 40x, NA 1.2 water immersion objective (Carl Zeiss, Jena, Germany). A Nd:YAG solid state laser was used for excitation of SRB at 532 nm. The fluorescence that passed through an appropriate dichroic beam splitter and a long-pass filter was imaged onto a 50-μm core fiber coupled to an avalanche photodiode (PerkinElmer Optoelectronics, Fremont, CA). The signal from an output was correlated by a correlator card (Correlator.com, Bridgewater, NJ). The data acquisition time was 30 s. The experimental data were obtained under stirring conditions which increased the number of events by about three orders of magnitude thus substantially enhancing the resolution of the method. Concentrations of BACE and chlorin e_6_ (about 100 nM) were used to produce the count rate of 100 kHz. For peak intensity analysis fluorescence traces with the sampling time of 25 μs were analyzed using WinEDR Strathclyde Electrophysiology Software designed by J. Dempster (University of Strathclyde, UK). The software, originally designed for the single-channel analysis of electrophysiological data, enables one to count the number of peaks (*n(F>F*_*0*_)) of the FCS signal having amplitudes higher than the defined value (*F*_*0*_) [8,58]. A program of our own design with a similar algorithm (coined Saligat; provided on request) was also used.

### Potassium leakage

Potassium concentration was measured with the help of a K^+^-sensitive electrode (NIKO-ANALIT, Moscow, Russia) in the medium of 100 mM choline chloride, 5 mM MOPS, рН 7.4.

### Confocal laser scanning microscopy of photosensitizer distribution in bacterial cells

To study photosensitizer distribution in *B*. *subtilis* cells, we used confocal laser scanning microscopy (CLSM). Bacteria cell suspensions were incubated with photosensitizers for 15 min in the minimal volume of PBS on coverslips in a Recon camera of an inverted microscope. Then the mounting medium—a mixture of 20% aqueous gelatin solution with an equal volume of glycerol—was added. Before using, the glycerol-gelatin mixture was heated to 50–60°C for 7 min, then allowed to cool to 37°C and quickly added to a coverslip with bacteria. Digital images were acquired using an Axiovert 200M LSM-510 META microscope (Carl Zeiss AG, Germany). The confocal images were recorded with a Plan-Apochromat 100x/1.4 Oil Ph3 objective. Fluorescence of photosensitizers was excited with a 633 nm He–Ne laser, and emission was detected with a 650–710 nm band pass filter.

### Detection of cell membrane integrity

Cell membrane integrity after illumination of bacteria in the presence of a photosensitizer was estimated using propidium iodide (PI). Bacterial cells were incubated for 15 min with 30 nM PI and then examined with a Nikon Eclipse Ti-E confocal laser scanning microscope with a Nikon Eclipse Ti-E A1 laser-scanning confocal system and a Plan Apo 20x/0.75 objective. Images were captured for 25 fields of views for each sample, and the number of cells per field of view was counted, as determined by PI staining and differential interference contrast microscopy (DIC).

### Statistics

All data are presented as means ± standard deviations as a result of 3–5 experiments.

## Results and Discussion

**Fig 1:**
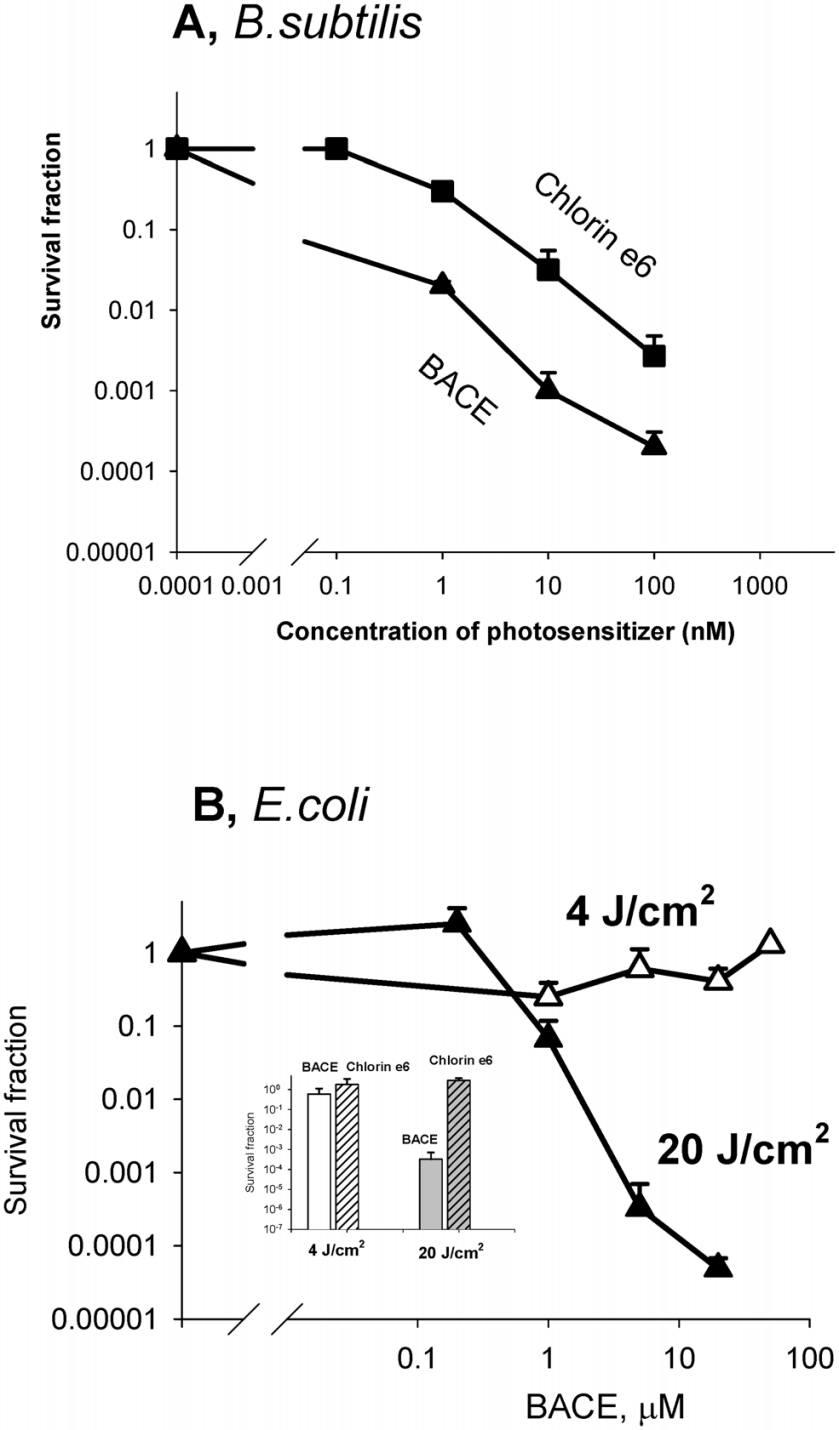
**A. Dose dependence of phototoxicity of BACE and chlorin e**_**6**_ **towards *Bacillus subtilis*.** Cells were incubated with the photosensitizers for 10 min in the dark and illuminated with red light at 4 J/cm^2^. Colony-forming units (CFU) were determined by serial dilution-agar plating method. The cell surviving fraction was evaluated as CFU experiment / CFU control. **B. Dose dependence of phototoxicity of BACE towards *E*. *coli*.** Cells were incubated with the photosensitizers for 10 min in the dark and illuminated with red light at 4 J/cm^2^ or 20 J/cm^2^. Photosensitizer concentration in the insert was 5 μM.

**Fig 2:**
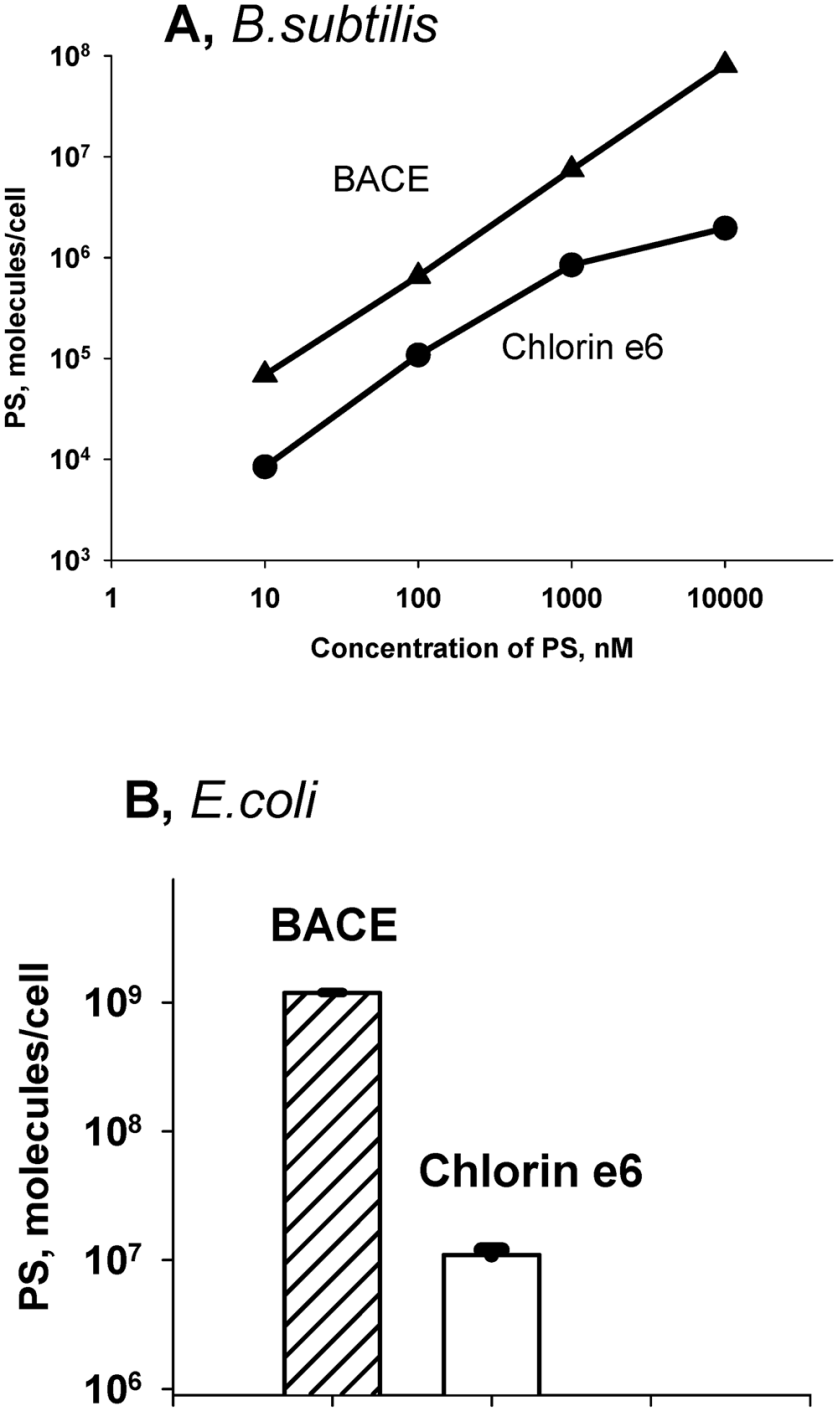
Uptake of BACE or chlorin e_6_ by *B*. *subtilis* (A) and *E*. *coli* (B) cells. Cells were incubated for 10 min in the dark with the indicated amount of a photosensitizer. The pellet obtained after centrifugation was treated with 0.1 M NaOH / 1% SDS. The photosensitizer concentration was determined from its fluorescence by using a calibration curve for the solutions of different concentrations in 0.1 M NaOH / 1% SDS.

Fig 1A displays the dependence of the survival of *B*. *subtilis* cells after photodynamic treatment on the concentration of photosensitizers, as measured by the conventional colony-counting method. It is seen that BACE at a concentration of 10 nM was substantially more effective in provoking PDI of *B*. *subtilis* than chlorin e_6_, which correlated with the corresponding difference (about two orders of magnitude) in the accumulation of BACE and chlorin e_6_ by *B*. *subtilis* cells (Fig 2A). It is worth mentioning that here we meant the increased association of the photosensitizer with bacterial cells without indication of its localization on the surface or inside cells. Bearing in mind that BACE and chlorin e_6_ were reported to have close values of the quantum yield of generation of singlet oxygen [8], with the latter being the key agent in the BACE photosensitizing activity in model systems [8,58], the increased photodynamic potency of BACE compared to chlorin e_6_ could be related to the enhanced accumulation of the boronated photosensitizer by bacterial cells.

In the PDI experiments with the gram-negative bacteria *E*. *coli*, chlorin e_6_ was completely inactive at a concentration of 5 μM (Fig 1B, insert), by contrast to its impact on the gram-positive species *B*. *subtilis* (Fig 1A), which was in line with the conclusion made by Zorin and coauthors [20] that the effectiveness of chlorin-mediated photoinactivation of *E*. *coli* is 100-200-times lower than that of *B*. *subtilis*. Our observations also agree with the data obtained for chlorin e_6_ in [24,53]. When applied with the illumination of 4 J/cm^2^, BACE was also ineffective to photosensitize PDI of *E*. *coli* (Fig 1B). However at 20 J/cm^2^, BACE appeared to effectively suppress the colony-forming activity of *E*. *coli* beginning from 1 μM (Fig 1B), in contrast to chlorin e_6_ (Fig 1B, insert). Similar to the case of *B*. *subtilis*, the enhanced photobactericidal activity of BACE compared to chlorin e_6_ with *E*. *coli* could also be attributed to the increased uptake of BACE by the bacterial cells (Fig 2B).

**Fig 3:**
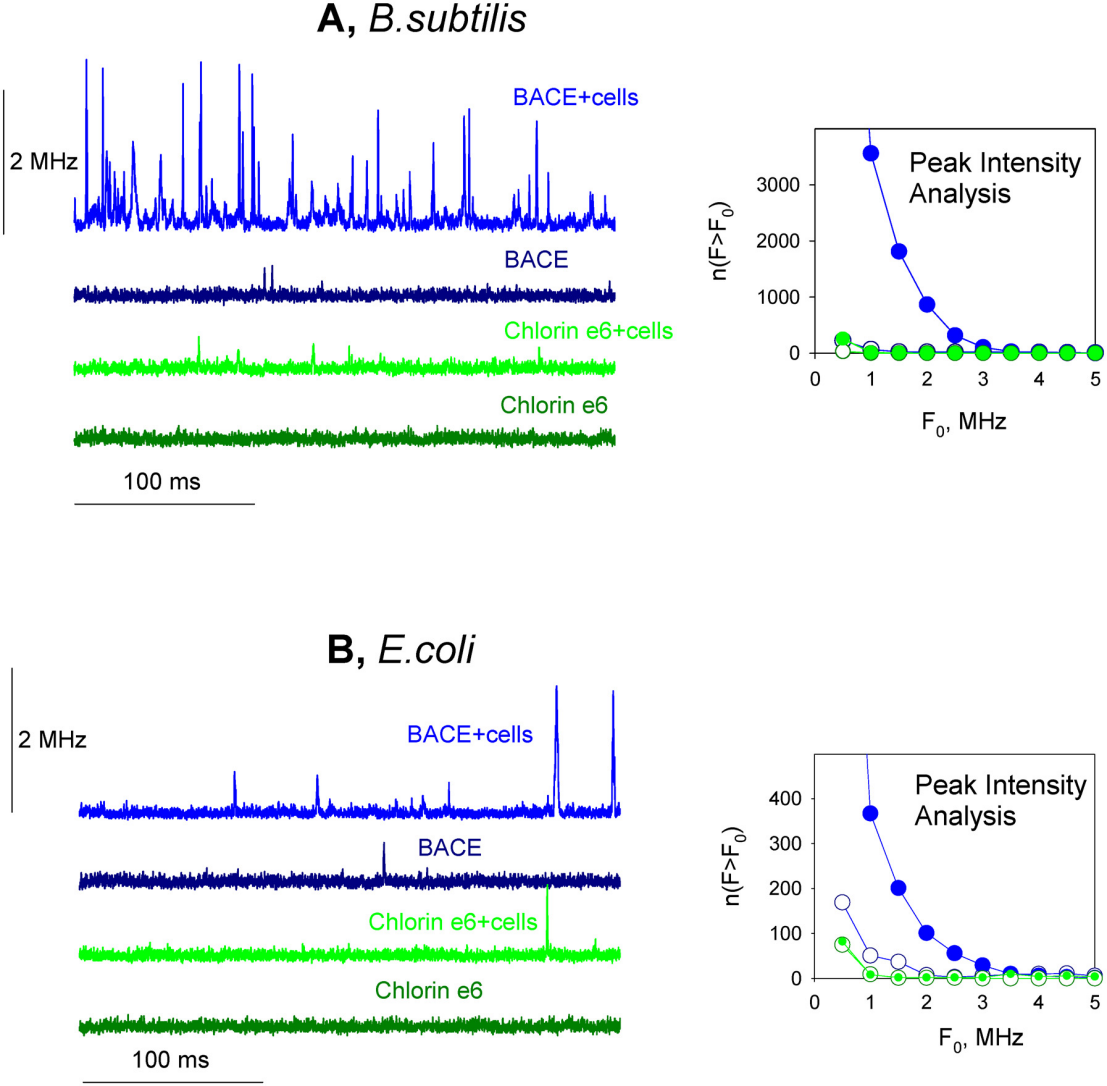
Accumulation of BACE and chlorin e_6_ by *B*. *subtilis* (A) and *E*. *coli* (B) cells monitored by FCS. Fluorescence intensity traces of photosensitizers were recorded with the FCS set-up in the presence or absence of bacterial cells (10^6^ per ml). *Inserts*: Corresponding dependences of the number of peaks with the fluorescence intensity *F* exceeding the threshold *F*_*0*_, *n(F>F*_*0*_ *)*, on the value of *F*_*0*_.

Bright fluorescence of chlorins enabled us to supplement the data on the macroscopic uptake of chlorins by bacterial cells (Fig 2) by FCS measurements of photosensitizer accumulation by single bacterial cells (Fig 3). The addition of *B*. *subtilis* cells to the solution of BACE resulted in the appearance of high-amplitude peaks in the fluorescence intensity traces recorded with an FCS set-up which reflected uptake of BACE by single bacterial cells (Fig 3A). The fluorescence peaks were much lower with chlorin e_6_, as compared to BACE. Similar results were obtained with *E*. *coli* cells (Fig 3B). Our data on the relationship between the photodynamic efficacy and the bacterial accumulation of BACE are in line with qualitative correlation between PDI of a panel of bacteria species using chlorin e_6_-polyethyleneimine conjugates [51] and their uptake by bacterial cells.

**Fig 4:**
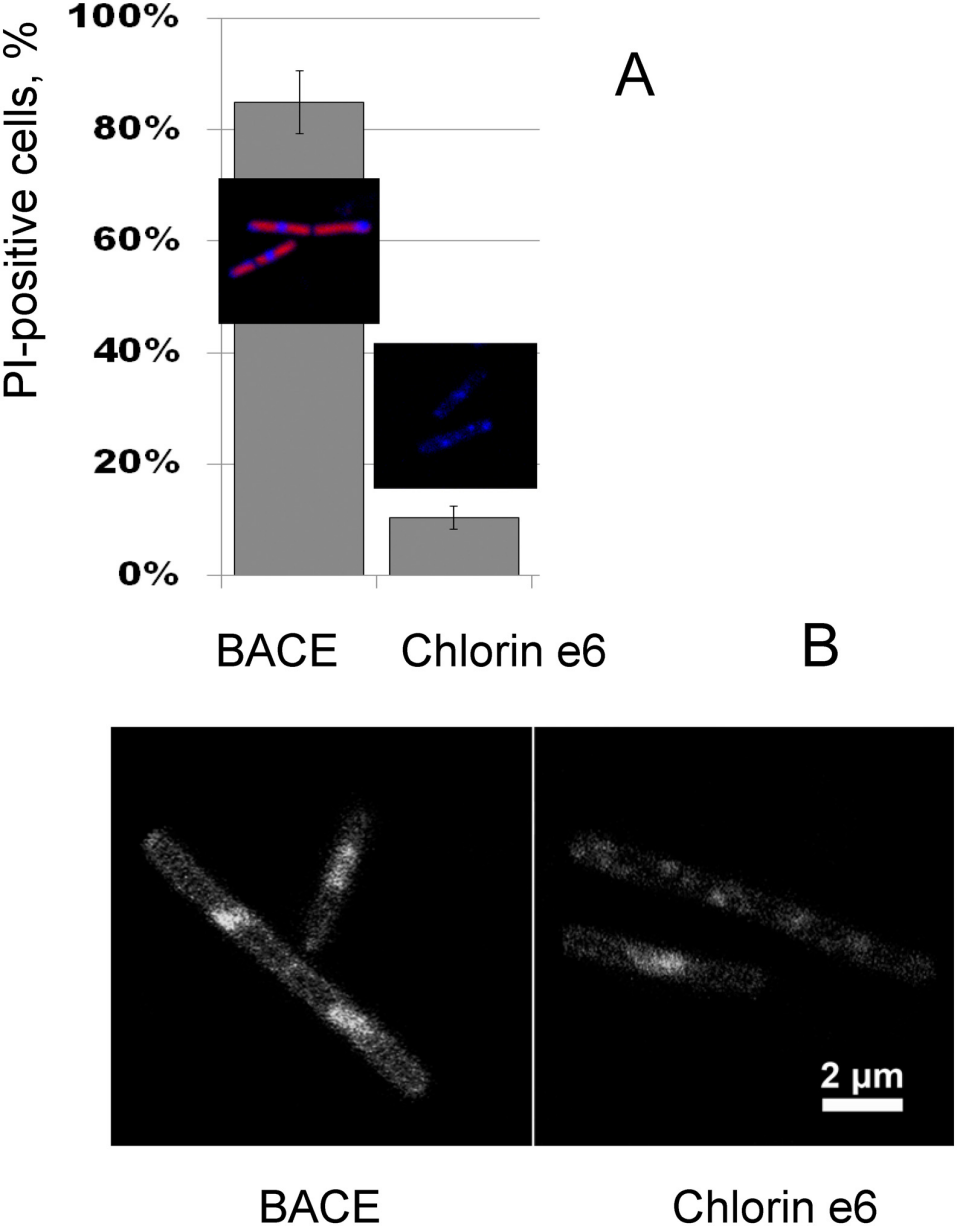
**A. Membrane integrity of BACE- and chlorin e**_**6**_ **-treated *B*. *subtilis* cells after illumination.** Photosensitizers are depicted in blue color, propidium iodide (PI)–in red color. Membrane integrity was assessed by counting PI-positive cells after illumination with red light at 4 J/cm^2^. **B. Intracellular localization of the photosensitizers measured by confocal laser microscopy.**

By applying CLSM to *B*. *subtilis* cells incubated with PI, we compared the light-induced effects on cell membrane integrity in the presence of BACE and chlorin e_6_. As seen from Fig 4A, the percentage of cells permeable to PI was much higher after the photodynamic treatment with BACE, than with chlorin e_6_, thereby indicating the involvement of membrane damage in the PDI of bacterial cells. This assumption was supported by CLSM images of BACE and chlorin e_6_ distribution in cells of *B*. *subtilis*, which revealed predominant localization of BACE on the surface of bacterial cells (Fig 4B). With *E*. *coli* the changes in PI permeability were negligible under these conditions (data not shown).

**Fig 5:**
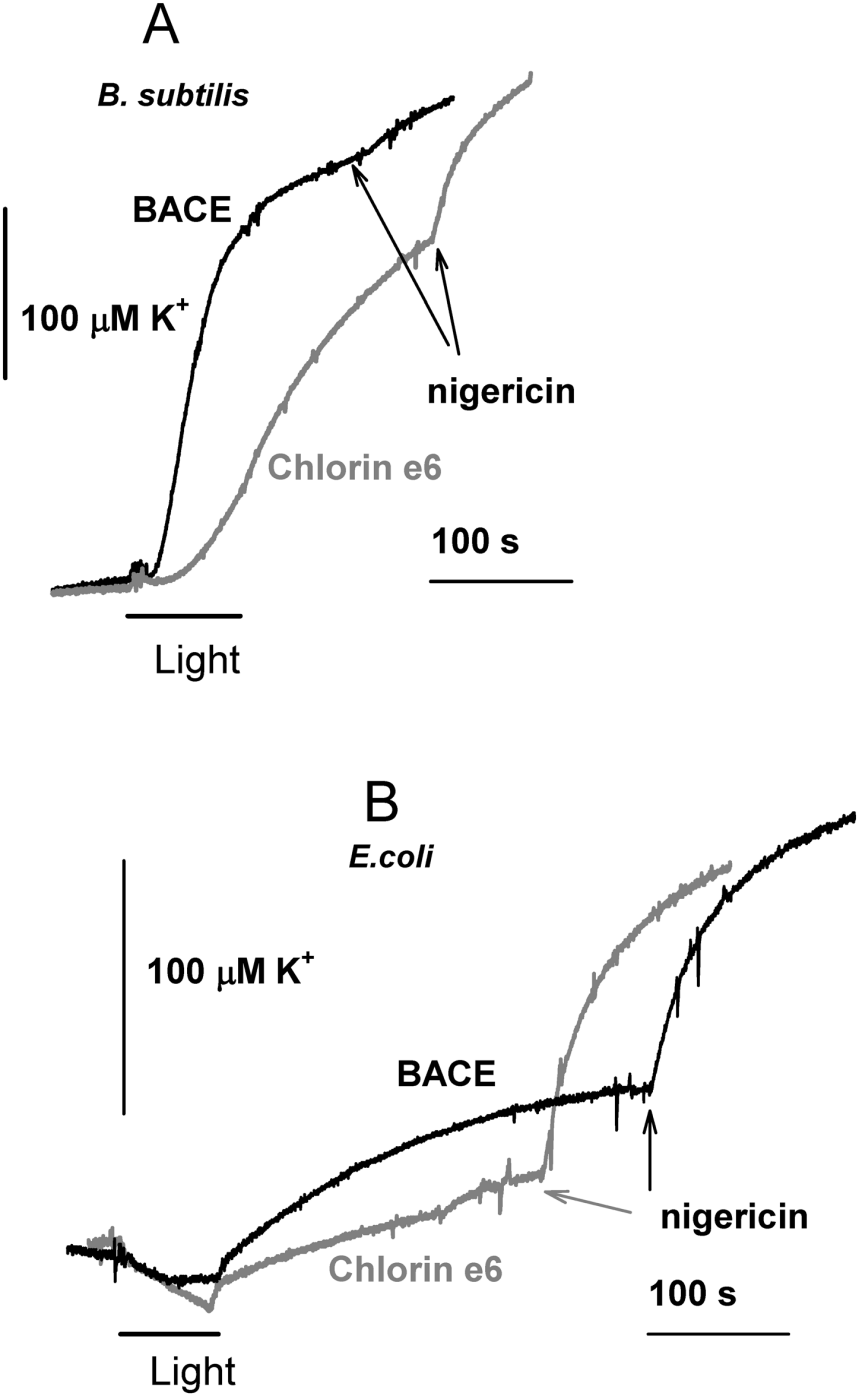
Potassium permeability of BACE- and chlorin e_6_-treated *B*. *subtilis* (A) and *E*. *coli* (B) cells after illumination measured by a potassium-selective electrode. Cells were incubated with photosensitizers for 10 min prior to illumination (4 J/cm^2^). Nigericin (1 μM) was added at the end of each recording to induce full potassium efflux.

To further investigate the membrane damage in the course of BACE-mediated aPDI, we measured its impact on the potassium leakage from bacterial cells [59]. As seen from Fig 5A, illumination (4 J/cm^2^) of *B*. *subtilis* cell suspension in the presence of the photosensitizers resulted in significant stimulation of potassium leakage from cells, with BACE being more effective than chlorin e_6_, whereas noticeable potassium leakage was not observed with *E*. *coli* cells under these conditions (Fig 5B).

**Fig 6:**
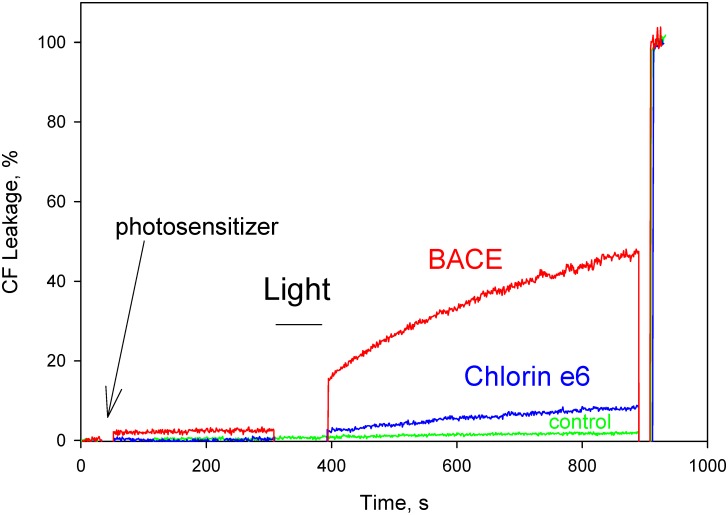
Photodynamic action on liposomes made from *E*. *coli* total lipids. Carboxyfluorescein leakage from liposomes induced by 1-min exposure to visible light (4 J/cm^2^) in the presence of BACE (red curve), chlorin e_6_ (blue curve), and a control without illumination and photosensitizers (green curve). The solution was 100 mM KCl, 10 mM Tris, 10 mM MES, pH 7.0. Lipid concentration was 5 μg/ml.

To compare the photodynamic potencies of the two photosensitizers in a model membrane system mimicking bacterial cell membranes, we examined the ability of BACE and chlorin e_6_ to sensitize the photodynamic leakage of the fluorescent dye CF from liposomes formed from *E*. *coli* total lipid extract with a high content (about 20%) of negatively charged lipids. Fig 6 shows that BACE at a concentration of 30 nM was much more effective than chlorin e_6_ in provoking the CF leakage under these conditions. Earlier chlorin e_6_-aided photodynamic leakage of CF from liposomes formed of neutral lipids was described in [60–62].

**Fig 7:**
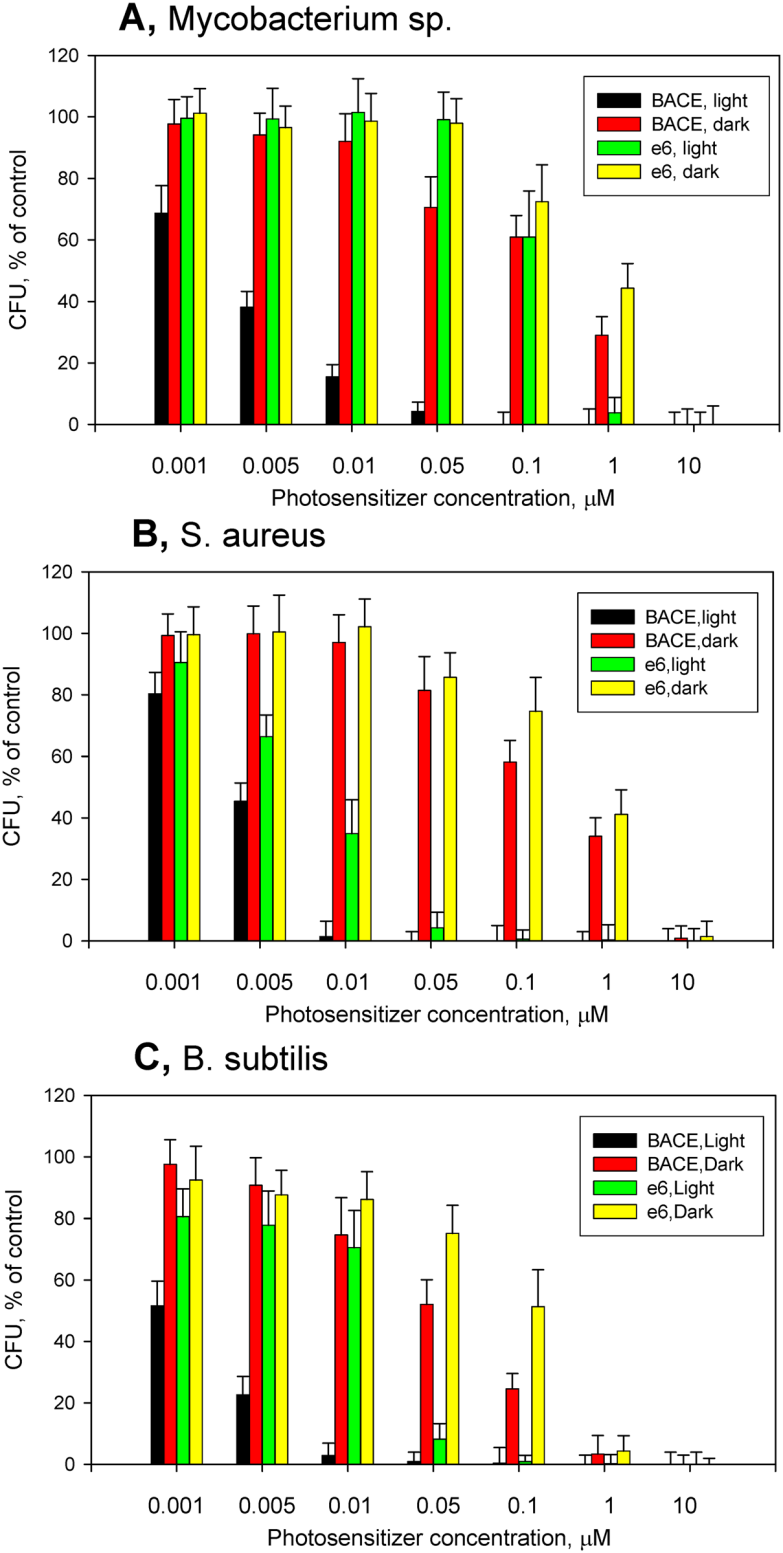
Phototoxicity and dark toxicity of BACE and chlorin e_6_ towards *Mycobacterium sp*. (A), *Staphyllococcus aureus* (B), and *Bacillus subtilis* (C) evaluated by CFU counts. Phototoxicity was determined upon illumination with red light at 4 J/cm^2^. The data shown are mean values ± standard deviations.

To compare sensitivity of different bacteria to PDI with BACE and chlorin e_6_, we determined cell viability for a series of bacteria species growing simultaneously in a 96-well plate under illumination in the presence of different concentrations of photosensitizers by using CFU counts (Fig 7). Remarkably, *Mycobacterium sp*. (Fig 7A) and *Staphyllococcus aureus* (Fig 7B) appeared to be highly sensitive to PDI with BACE (and less sensitive to PDI with chlorin e_6_), similar to *B*. *subtilis* (Fig 7C). The most striking difference in sensitivity to BACE and chlorin e_6_ was observed with *Mycobacterium sp*. (Fig 7A). *E*. *coli* were resistant to PDI in this system (data not shown), unless the concentration of BACE was increased to 50 μM, which exhibited dark toxicity against these bacteria. By contrast, with *Mycobacterium sp*., *S*. *aureus* and *B*. *subtilis*, dark toxicity of BACE was about two orders of magnitude lower than its phototoxicity (Fig 7).

The focus of the paper was to compare photodynamic efficacies of BACE and chlorin e_6_ under mild illumination conditions, i.e. at 4 J/cm^-2^. Under these conditions BACE appeared to be effective with a panel of Gram-positive bacteria and ineffective with Gram-negative bacteria, whereas for killing the latter illumination at 20 J/cm^-2^ was required. Therefore, to gain insight in the mechanism of the photobactericidal effect of BACE, we examined its impact on membrane integrity and performed leakage experiments also under mild illumination conditions.

**Fig 8:**
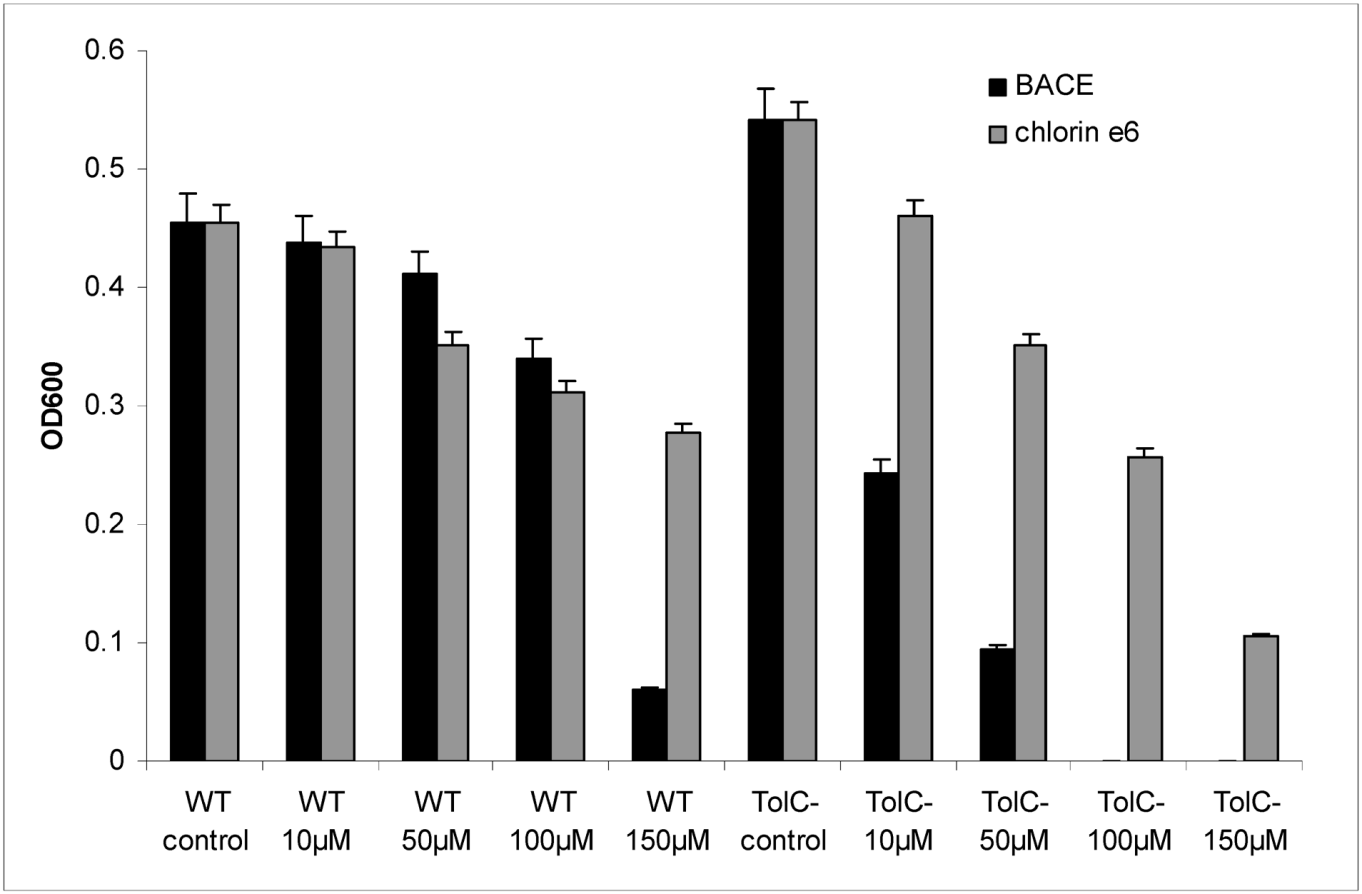
Dark toxicity of BACE and chlorin e_6_ towards WT *Escherichia coli* and the *E*. *coli* Δ*tolC* mutant. Chlorin e_6_ or BACE (100 nM—50 μM) were added to bacterial cultures (1–5*10^6^ cells/ml), placed in 96-well plates. Cell density was determined by absorbance at 600 nm. After that bacteria were allowed to grow in the dark within 21 hours and cell density was measured again.

Unlike chlorin e_6_, BACE showed increased dark toxicity with the *E*. *coli* Δ*tolC* mutant [55], deficient in TolC-requiring multidrug efflux transporters [63], compared to the wild-type strain (Fig 8). Similar difference was found earlier for phenothiazinium antimicrobial photosensitizers [64]. The results obtained with the *E*. *coli* Δ*tolC* mutant indicate that BACE is a substrate of *E*. *coli* multidrug resistance pumps. To support this assumption, we examined dark toxicity of BACE with the *E*. *coli* Δa*crE* mutant, derived by a single deletion of a*crE* from the same parent strain as the Δ*tolC* mutant. In *E*. *coli*, AcrE along with AcrB, AcrD, AcrEF, MdtABC, and MdtEF belong to resistance-nodulation-cell division (RND) family of multidrug efflux transporters that require interaction with TolC to function [65]. In fact, the *E*. *coli* Δa*crE* mutant displayed rather poor sensitivity to BACE (data not shown). Hence, the multifunctional outer membrane channel TolC is required for BACE efflux from *E*. *coli* cells.

In conclusion, BACE, a photosensitizer of the carboranyl-chlorin type, was shown here for the first time to be highly effective in killing a series of Gram-positive bacteria, with outer cell structures being most likely the major target of the photodynamic action. The enhanced efficacy of BACE as an antimicrobial photosensitizer with respect to chlorin e_6_ could be explained by the increased accumulation of the boronated derivative by bacterial cells.

## Abbreviations

PDI: photodynamic inactivation
aPDI: antimicrobial photodynamic inactivation
BNCT: boron neutron capture therapy
PS: photosensitizer
PI: propidium iodide
CF: 5(6)-carboxyfluorescein
BACE: chlorin e_6_ 13(1)-N-{2-[N-(1-carba-closo-dodecaboran-1-yl)methyl]aminoethyl}amide-15(2), 17(3)-dimethyl ester
CFU: colony forming unit
CLSM: confocal laser scanning microscopy
FCS: fluorescence correlation spectroscopy
